# The perceived influence of fathers' and non-gestational parents' experiences of postnatal depression on their relationship with their child and partner: a systematic review of qualitative studies and meta-synthesis

**DOI:** 10.3389/fpsyg.2026.1840073

**Published:** 2026-06-29

**Authors:** Jessica Raphael, Emily Eisner, Anja Wittkowski

**Affiliations:** 1 Division of Psychology and Mental Health, School of Health Sciences, The University of Manchester, Manchester, United Kingdom; 2 Greater Manchester Mental Health NHS Foundation Trust, Manchester, United Kingdom; 3 The Perinatal Mental Health and Parenting (PRIME) Research Unit, Department of Research and Innovation, Manchester, United Kingdom; 4 Manchester Academic Health Science Centre, Manchester, United Kingdom

**Keywords:** bonding, paternal, PND, PPD, qualitative, relationship dynamics

## Abstract

**Introduction:**

The role of the father/non-gestational parent has been found to be important in child development and wellbeing, but their mental health in the postnatal period is under-researched. Thus, this systematic review aimed to explore how postnatal depression was perceived to affect familial relationships.

**Methods:**

A systematic review of qualitative literature was conducted, and the Preferred Reporting Items for Systematic Review and Meta-Analysis guidelines were followed. Nine databases were searched for terms associated with fathers/non-gestational parents, postnatal depression, and qualitative and mixed method studies. Studies that met inclusion criteria were quality assessed using the Critical Appraisal Skills Program (CASP). Data were thematically synthesized.

**Results:**

Thirteen studies met the inclusion criteria. The methodological quality of all included papers was moderate to high. Following thematic synthesis, six themes and nine subthemes were identified, illustrated in a conceptual model. The main themes included 1) co-existing without connection, 2) withdrawing from others, 3) prior perceptions and expectations influencing view of self, 4) conditional connection with baby, 5) the dutiful parent, and 6) the role of partnership, validation and time for self-care as protective relationship factors.

**Conclusions:**

Relationship dynamics often worsened between fathers or non-gestating mothers with postnatal depression and their partners after the baby's birth. Participants with postnatal depression who experienced relational conflict with partners often expressed a mismatch between their parenting ideals and their actions. Fathers and non-gestating mothers reported a loss of identity and feelings of resentment when they carried out caregiving tasks dutifully but without emotional connection. When fathers carried out tasks in this way, they tended to avoid spending time at home, reinforcing their separation from the mother and their baby and increasing feelings of jealousy toward the mother–infant dyad. Non-gestational mothers did not express using avoidance as a coping strategy, highlighting a difference in the way men and women cope with difficulties in the family. Participants appeared to express a high internal locus of control, internalizing blame for their baby's negative reactions (e.g., crying or difficult to settle) and interpreting these responses as evidence that the baby disliked them, leading them to conclude they were a bad parent.

## 1 Introduction

Postnatal depression is an episode of minor or major depression in the postnatal period (O'Hara and McCabe, 2013). Whilst there has been a significant amount of research examining the prevalence of postnatal depression in mothers (and thus one side of the parental dyad), with a recent meta-regression analysis finding postnatal depression in 17.22% of the world's population (Wang et al., 2021), fewer studies have focused on the “other” parent. Fathers can also experience depression following the birth of their child, with 13%−26% of fathers reporting depression 3–6 months post-birth (Paulson and Bazemore, 2010; Rao et al., 2020) and 5%−10% of men in England experiencing depression in the perinatal period (Paulson and Bazemore, 2010). Similarly, in recent years with developments in *in vitro* fertilization, there is a growing number of same-sex parent households, with 217,000 same-sex couple families in the United Kingdom (UK) in 2022 (Just Like Us, 2024). Lesbian non-gestating mothers have expressed experiencing postnatal depression (McKelvey, 2014), which, according to Wojnar and Katzenmeyer (2014), has gone unnoticed and unaddressed by healthcare providers. Likewise, in a sample of 500 men, 32.4% of non-biological fathers in same-sex families have symptoms of depression in the postnatal period (Jianzheng, 2024). Whilst the identification of postnatal depression in mothers is a priority in perinatal healthcare settings, the “other” parent is not routinely considered despite calls in the UK for a family approach to perinatal mental health care (Darwin et al., 2021).

The first few years of a child's life are important because they play a large role in a child's development (Hoskings and Walsh, 2010; Leadsom et al., 2013), with parental mental health difficulties negatively impacting children's own distress trajectories (Kamis, 2021). Consequently, there have been initiatives aimed to improve parental mental health to enable better outcomes for children (Her Majesty's Government, 2021). Studies exploring the impact of maternal postnatal depression on children found that children develop insecure attachment styles (Edhborg et al., 2001; Martins and Gaffan, 2000; Murray, 1992; Teti et al., 1995), have poorer emotional outcomes, such as reduced joy (Edhborg et al., 2001) and poor social-emotional development (van der Voort et al., 2014). However, few studies have explored if there is a similar effect for fathers and non-gestating parents (Paulson and Bazemore, 2010; Hogg, 2014; Sanger et al., 2015). Despite limited exploration in this field, preliminary research does indicate negative effects of paternal depression on families, leading to behavioral difficulties and poor social, emotional and cognitive development (Dachew et al., 2023; Fletcher et al., 2011; Gutierrez-Galve et al., 2015; Ramchandani et al., 2008; Wanless et al., 2008), and poorer neurodevelopmental outcomes (Ashraf et al., 2023). These findings highlight the importance of assessing both parents for depression following birth so that depression can be identified early, and parents can be offered appropriate psychological support quicker to help prevent adverse outcomes in children (Pilkington et al., 2017).

The UK's National Institute for Health Care Excellence (2020) recommends using the Edinburgh Postnatal Depression Scale (EPDS; Cox et al., 1987) to assess for postnatal depression in pregnant women and gestational parents. Whilst Edmondson et al. (2010) found moderate validity when using EPDS with fathers, there is no current recommended tool to assess postnatal depression in fathers or non-gestational parents. It is, therefore, possible that postnatal depression in these parents goes undetected and they go without support. This barrier to accessing care can be compounded by the self-stigma faced by fathers to conform to western societal norms of being masculine and thus not talking about their emotions (Reay et al., 2023), but also by non-gestating parents in same-sex couples, in relation to the uncertainty of their role in the perinatal period (Goldberg et al., 2020; Haughland et al., 2023).

To address the gap in the research, there have been several systematic reviews that explored and thus highlighted the prevalence of postnatal depression in fathers (Cameron et al., 2016; Dhanpal and Shil, 2024; Paulson and Bazemore, 2010), investigated the availability of screening depression for fathers during the perinatal period (Álvarez-García et al., 2024; Berg et al., 2022; Mancini et al., 2025; Schöch et al., 2024), and examined the barriers fathers experienced when accessing interventions for emotional support in the perinatal period (Mancinelli and Filippi, 2025). Likewise, several systematic reviews have been conducted to explore the effects of paternal depression on child and adolescent outcomes (Dachew et al., 2023; Le Bas et al., 2025; Scarlett et al., 2024; Sweeney and MacBeth, 2016), a concept analysis of paternal perinatal depression (Chen et al., 2023), and the effects of paternal depression on parenting behaviors (Cheung and Theule, 2019; Wilson and Durbin, 2010). However, many of these reviews were quantitative and focussed on depression in fathers broadly (not specifically here postnatal depression) and none include non-gestational parents from same-sex families.

Qualitative research helps to enrich our understanding of patient, staff and provider perspectives in relation to health behaviors, motivations and expectations (Bazen et al., 2021). Additionally, updated guidelines by the Medical Research Council and National Institute for Health Research (Skivington et al., 2021), which aim to facilitate complex intervention design, stipulate the need to consider the development and identification stage of designing complex interventions through various means, including the exploration of stakeholder views to inform the evaluation of research. To date, there has been one qualitative systematic review, which explored fathers' experiences of perinatal depression (Davenport et al., 2022). Six themes were synthesized from nine included papers relating to poor mental health literacy in relation to paternal perinatal depression, relationships as comforting and distressing, avoiding paternal perinatal depression, fathers remaining silent for fear of judgement, lack of non-targeted support preventing help-seeking, and the challenges of fatherhood. However, this review included only two papers focusing solely on depression, with a particular focus on how fathers experienced postnatal depression, rather than exploring the perceived impact on their family (partner and child). Thus, this systematic review and meta-synthesis aimed to explore the parent-reported influence of postnatal depression in fathers and non-gestating parents on their relationships with their child and partner/spouse.

## 2 Methods

This meta-synthesis, registered with the PROSPERO database (https://www.crd.york.ac.uk/PROSPERO/view/CRD420261309428), was conducted in line with the Preferred Reporting Items for Systematic Reviews and Meta-Analysis (PRISMA) guidelines (Page et al., 2021).

### 2.1 Search strategy

The search strategy was developed using the PICo (Population, Phenomena of Interest, and Context) framework, which was adapted to include qualitative methodologies (e.g., “qualitative,” “thematic,” etc.) to reduce the number of titles and abstracts that required screening to a manageable number (Stern et al., 2014). Medical Subject Heading (MeSH) terms were utilized to capture synonyms and Boolean operators (“AND,” “OR”) were used to combine terms (see Table 1). Nine databases, deemed most relevant for psychological and maternity literature, were searched from inception until December 2025, on the following platforms: OVID (Allied and Complementary Medicine, AMED; Excerpta Medica database, EMBASE; Maternity and Infant Care; Medical Literature Analysis and Retrieval System Online, MEDLINE; PsycINFO), ProQuest (Applied Social Sciences Index and Abstracts, ASSIA; British Nursing Index, BNI and archive), EBSCOHost (Cumulative Index of Nursing and Allied Health Literature, CINAHL Plus), and Clarivate (Web of Science). No date limiters or language restrictions were used. Additional studies were identified by scanning the reference lists of relevant (qualitative or mixed method) literature reviews and eligible articles, and by forward citation searching.

**Table 1:** Search terms, strategy, and limits.

Search strategy number	PICo framework	Examples of words searched for
1	Population	(Father^*^ OR Paternal OR non-gestating parent^*^ OR non-birthing parent^*^ OR partner^*^ OR co-parent^*^ OR second parent OR dad OR non-gestat^*^ OR Gay parent^*^ OR Lesbian parent^*^ OR Homosexual parent^*^ OR Same sex parent^*^ OR LGBT^*^ parent^*^ OR Men OR Male)
2	Phenomena of Interest	(postpartum depress^*^ OR post-partum depress^*^ OR postnatal depress^*^ OR post-natal depress^*^ OR perinatal depress^*^ OR peri-natal depress^*^ OR perinatal mental health^*^ OR Postpartum OR Post-partum OR Post-natal OR Postnatal OR Perinatal OR Peri-natal OR PND OR Depression in post^*^ OR Mental health)
3	Context	(Parent-child relationship^*^ OR Father infant bonding OR attachment OR family dynamics OR family functioning OR co-parenting relationship OR relationship^*^ OR child development OR infant outcomes OR parental stress OR family outcomes)
4	Methodology	(Qualitative OR synthesis OR ethnography OR thematic^*^ OR content analys^*^ OR narrative^*^ OR discourse analys^*^ OR grounded theory OR IPA OR Interpretive Phenomenological Analys^*^ OR Concept analys^*^)
5	1 AND 2 AND 3 AND 4	
	Limits: abstract and title.	

### 2.2 Eligibility criteria

Paper inclusion criteria were: 1) qualitative or mixed methods studies with a qualitative component (e.g., a survey open text box) in which quotes could be extracted for analysis with no restrictions on language; 2) studies that used a sample of fathers or non-gestational parents with self-reported or diagnosed postnatal depression; additionally, due to the small number of available papers focusing solely on postnatal depression, studies that included a study sample of fathers/non-gestational parents with postnatal depression in addition to other mental health difficulties were included; and 3) studies in which participants described the perceived influence of postnatal depression on the relationship with the mother/gestational parent/child/family unit.

Papers were excluded if they were a) quantitative studies because their ability to address parent-reported influence of postnatal depression on relationships can be limited, b) qualitative studies using secondary data that were not reported by the participant (e.g., clinical notes), c) literature reviews, d) conference abstracts for which no full eligible paper was retrieved, and e) clinical trial registration and protocols of planned studies. Additionally, studies were excluded when the sample included couples whereby the father/non-gestational parent did not also have postnatal depression, mothers who had partners with postnatal depression, single fathers/non-gestational parents, and fathers/non-gestational parents without postnatal depression; for example, when studies described “stress” or symptoms of depression, whilst not referring explicitly to “depression,” or when they referred to depression but not in the postnatal period.

### 2.3 Study selection

Studies meeting eligibility criteria were imported into Endnote (version 21). After duplicate references were identified and deleted, the first author screened the remaining title and abstracts for relevance, using the inclusion/exclusion criteria. A second researcher, unconnected to this review, independently screened 25% of randomly selected records. When both researchers agreed on exclusions, those records were discarded. The inter-rater agreement at the screening stage was almost perfect (99.24% agreement, κ = 0.92). At the second stage, the first author independently assessed all the full texts against the inclusion criteria, and the second researcher independently assessed 30% which were randomly selected. When both researchers agreed on exclusion, full texts were discarded and exclusion reasons recorded. The inter-rater agreement at full text screening was substantial (96.81% agreement, κ = 0.65). Any disagreements regarding papers to be included were discussed and resolved by the whole review team. All three authors agreed on the inclusion of the final papers for this review.

### 2.4 Quality assessment

Using the ten-item Critical Appraisal Skills Program (Critical Appraisal Skills Programme UK, 2024) checklist, all eligible qualitative studies were assessed for quality of methods. For example, if the research design was sufficient to answer the research question and if data analysis was sufficiently rigorous. A second researcher independently assessed 100% of the included studies, any disagreements were resolved through discussion between both reviewers.

Although the CASP was designed to be rated qualitatively by answering “Yes,” “No,” or “Cannot tell” to each of the ten questions, we opted to supplement this approach with a numerical system (e.g., “Yes” = 1, “Partially agree” = 0.5, and “No” = 0; see Harries et al., 2023; Butler et al., 2020). This approach allowed categories of methodological quality to be defined as high (9–10), moderate (6–8) and low ( ≤ 5), as stipulated by Butler et al. (2020), in addition to helping with comparison across other studies and providing an additional layer of transparency.

### 2.5 Data extraction and analysis

Full texts of identified and selected papers were saved as documents and uploaded into NVivo (version 15). The results or findings sections of the papers were synthesized using thematic synthesis as described by Thomas and Harden (2008), utilizing three steps: 1) “line-by-line” coding of the text, 2) developing “descriptive themes” and 3) then creating “analytical themes.” Thematic synthesis was selected because it was the most appropriate to explore new meaning in existing qualitative research and the method is considered appropriate for informing policy and practice (Barnett-Page and Thomas, 2009). Additionally, the process is clear and transparent and thereby easily replicable. Quotations from the original participants were synthesized; when there were minimal quotes in an included study, author interpretations from the results section were also synthesized. As we expected to synthesize papers with diverse samples (e.g., fathers and non-gestational parents) and presentations (e.g., postnatal depression or postnatal depression with other diagnoses), we took the decision to approach analysis as follows: papers were initially analyzed separately by group (e.g., first fathers, then non-gestational parents and, finally, diagnoses), before all included papers were reviewed as a whole to determine similarities across the whole dataset of studies and to develop the main themes and subthemes of the current review. Whilst only the first author extracted the data, the inclusion of papers and the analysis were discussed in regular team meetings and agreed by all authors.

### 2.6 Reflexivity statement

The first author was a trainee clinical psychologist with lived experience of postnatal anxiety and spouse paternal postnatal depression. The second author was a research psychologist with digital mental health expertise and lived experience of postnatal depression. The third author was an academic and clinical psychologist and expert in perinatal mental health, including the therapeutic support of mothers and babies and other family members in inpatient psychiatry and community mental health settings.

Together, the team ensured data were evaluated from a position which considered clinical and health implications whilst also considering the potential power imbalance that might have been present between researchers and participants (McDonald, 2021). As a team, we tried to be aware of not biasing interpretations toward euro-centric viewpoints, which was particularly important when considering relationships and bonds. As Keller (2018) critiqued attachment theory due to the perception that it has a western-specific monotropic understanding of relationships and parent styles, the research team used team discussions to reduce the likelihood of interpreting the data in a biased manner.

## 3 Results

### 3.1 Characteristics of included studies

**Figure 1:**
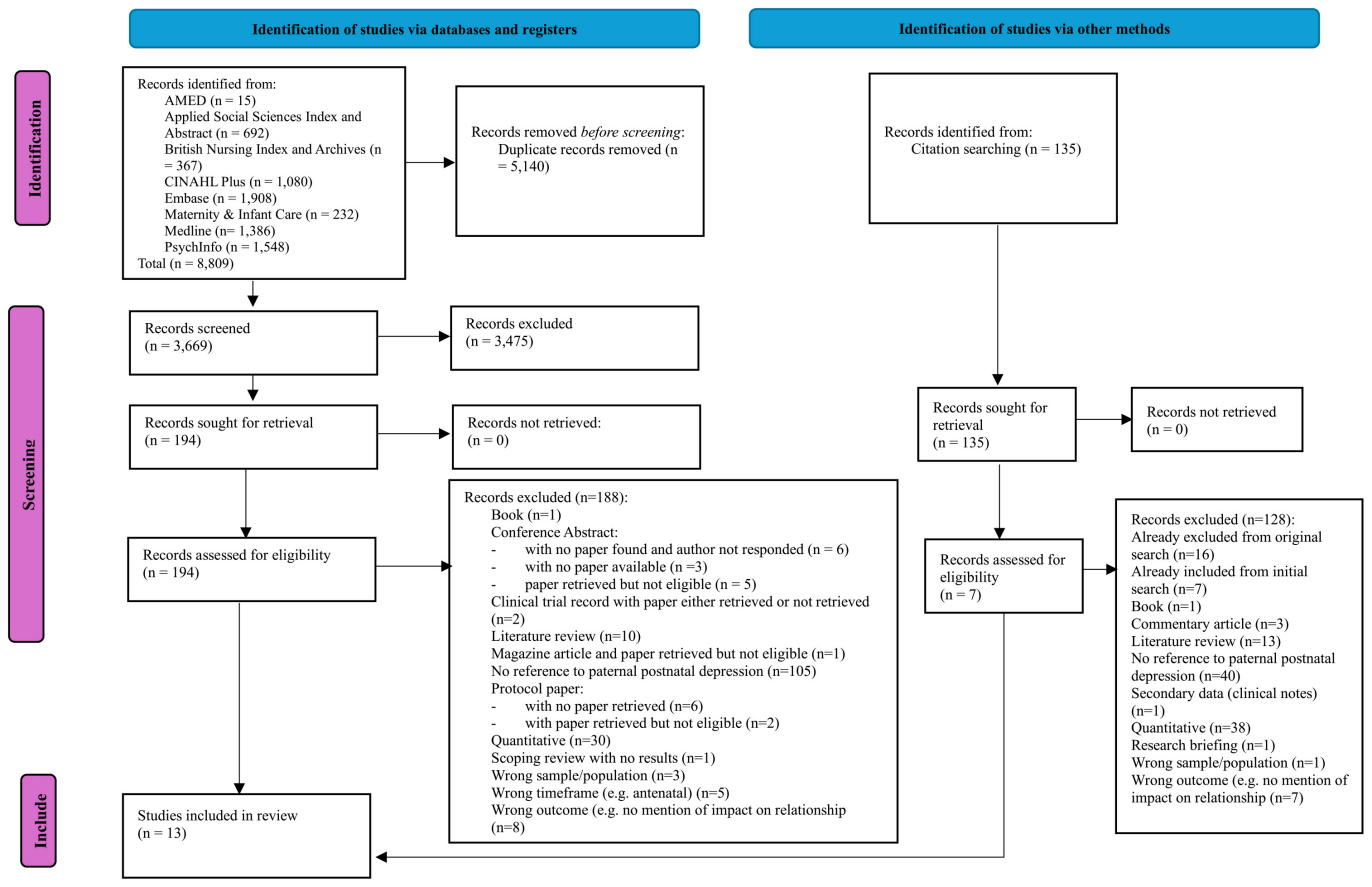
PRISMA flow diagram.

Of the 8,809 identified studies, following removal of duplicates, 3,804 studies were screened against eligibility criteria. From 306 full texts screened, 13 studies were identified and synthesized (see Figure 1). The studies were conducted in six countries (e.g., UK, USA, Denmark, Sweden, Philippines, and India). Despite not adding a date limiter, the studies were published between 2011 and 2025, highlighting that postnatal depression in fathers and non-gestational parents has been a relatively recent topic of research exploration. Table 2 presents an overview of the characteristics of the 13 included studies, with a minimum of 122 fathers or non-gestational parents with postnatal depression included across the 13 studies.

**Table 2:** Characteristics of the 13 included studies presented in reverse chronological order and grouped by diagnoses.

Number	Authors, year and location	Study aim	Number and participant sample	Diagnosis and verification	Common socio-demographic information (when provided)	Recruitment method	Data collection/analysis	Main theme titles
Studies including samples with a diagnosis of postnatal depression								
1.	Malik (2025), India	To explore the experiences of men with postnatal depression	*N* = 15 fathers	Postnatal depression diagnosed by medical professional in the past year (*n* = 15)	*Age of participants:* Ranged from 23 to 34 years old *Number of children* One (*n* = 7) Two (*n* = 6) Three (*n* = 2) *Age of children* Not reported *Marital status* Not reported *Education and/or employment status* Graduation (*n* = 9) Postgraduation (*n* = 6) *Ethnicity/nationality* Not reported	Psychiatric department via psychiatrists delivering postnatal depression treatment	Semi-structured interviews Colaizzi's seven-step approach (employed in Singh and Malik, 2022)	Four main themes (no sub-themes): 1) Men's silence in a relationship 2) Economic burden 3) Sleep deprivation 4) Enmeshment
2.	Schmitz (2025), USA	To explore the experiences of new fathers with postnatal depression	*N* = 10 fathers	Postnatal depression both self-reported and clinically diagnosed (*n* = 10) via a demographic's questionnaire	*Age of participants:* 18–25 (*n* = 2) 26–34 (*n* = 2) 35–45 (*n* = 6) *Number of children:* One (*n* = 8) >One (*n* = 2) *Age of children:* Not reported *Marital status:* Married (*n* = 6) Engaged (*n* = 2) Unmarried (*n* = 2) *Education and/or employment status:* Full-time (*n* = 7) Part-time (*n* = 1) Unemployed (*n* = 2) *Ethnicity/nationality:* Not reported	Flyers posted in local obstetricians, pediatricians and mental health providers. Shared on social media via the researchers Instagram and Facebook. Shared on Facebook parenting groups.	Semi-structured interviews Manen's hermeneutic phenomenological approach (van Manen, 1990)	Three main themes (no sub-themes): 1) Trauma 2) Depression 3) Societal role conflict
3.	Davenport and Swami (2023), UK	The lived experience of paternal postnatal depression in UK father's emotional experiences, working practices and relationships with partners and child.	*N* = 8 fathers	Postnatal depression (*n* = 8) Formal diagnosis from health professional (*n* = 3), PHQ-9 (Spitzer et al., 1999) scores of mild or moderate with no evidence of suicidality.	*Age of participants:* Age 27–41 years *Number of children:* One (*n* = 6), >One (*n* = 2) *Age of children:* Not reported *Marital status:* Long-term relationship and cohabiting (*n* = 6), living separately (*n* = 2) *Education and or/employment status:* Not reported *Ethnicity/nationality:* White British (*n* = 7) Asian (*n* = 1)	Social media (Twitter, Mumsnet, Dadsnet, Postgraduate Forum, Facebook, and the second author's university student Intranet. The study was also shared across local news outlets and via a local radio interview) and snowball sampling	Semi-structured interviews Interpretative Phenomenological Analysis (Smith et al., 2009)	Two main themes: 1) Fathers' emotional and embodied experiences of depression (five sub-themes) 2) PND and fathers' social environments (five sub-themes)
4.	Baral and de Guzman (2021), Philippines	To understand coping among depressed new fathers	*N* = 7 fathers	Postnatal depression (*n* = 7) Edinburgh Postnatal Depression Scale (Cox et al., 1987) and Gotland Male Depression Scale (GMDS; Rutz et al., 1995) 48–72 h post-childbirth.	*Age of participants:* Mean age 25.7 (SD = 4.34, range = 19–32) *Number of children:* Not reported *Age of children:* Not reported *Marital status:* Married (*n* = 7) *Education and/or employment status:* Not reported *Ethnicity/nationality:* Not reported	Screening 70 new fathers in 10 barangays of Cabanatuan City from the province of Nueva Ecija. Becks Depression Inventory was also used to exclude those with depression prior to childbirth.	Semi-structured interviews/Straussian Grounded Theory Design (Glaser and Strauss, 1967).	Four main themes creating the FEAR model: 1) Financial capability 2) Emotional response 3) Adaptability 4) Role transition
5.	Pedersen et al. (2021), Denmark	To explore the experiences of fathers with postnatal depression and understand barriers and facilitators to help-seeking	*N* = 8 fathers	Postnatal depression (*n* = 8) diagnosed by GP or psychologist as verbally confirmed by participant.	*Age of participants:* Ranged from 29 to 38 years old *Number of children:* One (*n* = 7) Two (*n* = 1) *Age of children:* 1 year old (*n* = 2) 2 years old (*n* = 3) 3 years old (*n* = 1) 5 years old (*n* = 3) *Marital status:* Not reported *Education and or/employment status:* Vocational (*n* = 6) Lower secondary (*n* = 1) Bachelor's degree (*n* = 1) *Ethnicity/nationality:* Not reported	Social media (Facebook, pregnancy and paternity groups, and forums for new and future parents)	Semi-structured interviews Interpretative Phenomenological Analysis (IPA; Pietkiewicz and Smith, 2014)	Two main themes: 1) Experiences of fatherhood (four sub-themes) 2) Help-seeking behavior (five sub-themes)
6.	Eddy et al. (2019), USA	To explore how fathers described their own experience of postnatal depression and what helps them	*N* = 27 fathers	Postnatal depression (*n* = 27) Self-reported on data sources utilized	Not collected	Data taken from blogs, chat rooms, book reviews, and interactive discussion forums.	Searched terms “depression in new gathers,” and “father depression after baby” and found forums: howisdaddydoing.org, citydadsgroup.com, thedaddycomplex.com and sub threads of reddit.com regarding postnatal depression in men Content analysis (Eysenbach and Till, 2001).	Six main themes: 1) Needing education 2) Adhering to gender expectations 3) Repressing feelings 4) Overwhelmed 5) Resentment of baby 6) The experience of neglect
7.	Darwin et al. (2017), UK	To examine the views and experiences of fathers who have perinatal psychological distress and understand what resources are accessible and acceptable	*N* = 19 fathers	Postnatal depression (*N* = 19) Postnatal scores: mean Patient Health Questionnaire-8 (PHQ-8; Kroenke et al., 2009) 4.8 (SD = 2.8, range = 0–10) Mean General Anxiety Disorder-7 (GAD-7; Spitzer et al., 2006) 4.0 (SD= 2.9, range 0–8) Mean Patient Health Questionnare-15 (PHQ-15; Kroenke et al., 2002) 4.0 (SD= 2.7, range = 0–10)	*Age of participants:* Mean age 22.1 years old (SD = 5.1, range = 25–44 years) *Number of children:* One (*n* = 14), >One (*n* = 5) *Age of children:* Mean age of youngest child 8.1 months (SD = 1.5, range = 5.1–9.8 months) *Marital status:* Married (*n* = 16) Cohabiting (*n* = 3) *Education and/or employment status:* Employed full time (*n* = 17) Employed part time (*n* = 1) Unemployed (*n* = 1) *Ethnicity/nationality:* White British (*n* = 18) White other (*n* = 1)	Born and Bred in Yorkshire (BaBY) eligible fathers sent information via the post.	Semi-structured interviews Thematic Analysis (Braun and Clarke, 2006)	Four main themes: 1) Legitimacy of paternal stress and entitlement to health professionals' support (three sub-themes) 2) Protecting the partnership 3) Navigating fatherhood (three sub-themes) 4) Diversity of men's support networks (three sub-themes)
8.	Edhborg et al. (2016), Sweden	To describe new fathers' experiences during the first year postnatal with depression 3–6 months postnatal	*N* = 19 fathers	Postnatal depression (*N* = 19) Median EPDS (Cox et al., 1987) 12.5 (range = 5–27) Median GMDS (Rutz et al., 1995) 13 (range = 3–32)	*Age of participants:* Mean age 36 years old (range = 25–52 years) *Number of children:* One (*n* = 9) Two (*n* = 8) Three and above (*n* = 2) *Age of children:* Mean age 9 months (range = 6–14 months) *Marital status:* Married (*n* = 12) Cohabiting (*n* = 7) *Education and/or employment status:* Secondary school (*n* = 4) University (*n* = 15) *Ethnicity/nationality:* Swedish (*n* = 17) Non-Swedish (*n* = 2)	All men in Stockholm who had become fathers November–December 2010 and May–June 2011who responded to questionnaires measuring depression 3–4 months postnatal. From these, 64 fathers who participated in an intervention with the first 30 being invited to take part in an interview and those who consented and could be contacted	Semi-structured interviews Content analysis (Graneheim and Lundman, 2004)	One main theme: 1) Loss of control and powerlessness (five sub-themes)
9.	Dallos and Nokes (2011), UK	To explore the experiences of first-time fathers with psychological difficulties following childbirth	*N* = 5 fathers with children < 1 years of age and no psychological distress to develop an interview schedule via a focus group *N* = 1 father with postnatal depression who had the interview using the interview schedule developed above	Postnatal depression (*n* = 1) diagnosed via EPDS (Cox et al., 1987)	Focus group (*n* = 5) *Age of participants:* 28–40 years *Number of children:* Not reported *Age of children:* < 1 years (*n* = 5) *Marital status:* Not reported *Education and/or employment status:* Mixture of professional and semiskilled backgrounds *Ethnicity/nationality:* Not reported Interview (*n* = 1) *Age of participant:* 40 years *Number of children:* Three who do not live with father One stepchild living with father One biological child living with father *Age of children:* 13 years old 5 months old *Marital status:* Married *Education and/or employment status:* Employed full time *Ethnicity/nationality:* Not reported	Parenting groups for fathers who developed the topic guide Clinical health psychology setting for father who took part in the interview	Focus group/Semi-structured interview Interpretative phenomenological analysis (IPA; Smith, 1999, 2004)	Two main themes: 1) Loss (one sub-theme) 2) Difficulties with adjustment (three sub-themes)
Studies including samples with mixed mental health diagnoses								
10.	Copland and Hunter (2025), UK	To explore the experiences of fathers with poor mental health during the perinatal period, and their perceptions of barriers and facilitators to mental health support	*N* = 8 fathers with poor mental health 6 months-2 years post birth	Anxiety/depression (*n* = 1) Anxiety (*n* = 3) Depression (*n* = 2) Anxiety/stress (*n* = 1) Stress (*n* = 1) Self-reported or diagnosed by healthcare professional (method of data gathering not reported)	*Age of participants:* Not reported *Number of children:* One (*n* = 5) Two (*n* = 3) *Age of children:* < 1 year (*n* = 5) 2 years (*n* = 3) 4 years (*n* = 2) 6 years (*n* = 1) *Marital status:* Partnered (*n* = 8) *Education and/or employment status:* Employed (*n* = 8) *Ethnicity/nationality:* Not reported	Adverts shared on social media and displayed in community groups and services.	Semi-structured interviews Thematic analysis (Braun and Clarke, 2006)	Three main themes: 1) Fathers are not the priority (two sub-themes) 2) The perinatal period is unique (three sub-themes) 3) Dad-specific support (two sub-themes)
11.	Howat et al. (2023), UK	To explore non-birthing mothers' experiences of perinatal depression and anxiety	*N* = 7 lesbian or bisexual mothers	Postnatal depression and/or anxiety (*n* = 7) Self-reported or medically diagnosed (method of data gathering Not reported)	*Age of participants:* Mean age 35 years (range 27–40 years) *Number of children:* Not reported *Age of children:* Mean age 13 months (range 2–36 months) *Marital status:* Married/civil partnership (*n* = 5) cohabiting (*n* = 2) *Education and/or employment status:* Full-time (*n* = 5) Part-time (*n* = 2) *Ethnicity/nationality:* White British (*n* = 6) White other (*n* = 1) *Sexual identity:* Lesbian (*n* = 6) Bisexual (*n* = 1)	Social media sites, voluntary and support organizations for LGBTQIA+ communities and for postnatal mental health.	Semi-structured interviews IPA (Larkin et al., 2021)	Six main themes (no sub-themes): 1) Parenting without 2) Changed relationship dynamics 3) Failure and inadequacy in role 4) Powerlessness and intolerable uncertainty 5) Legitimacy of (Di)stress as a non-birthing parent 6) Moving forward
12.	Atkinson (2023), UK	To develop a theoretical understanding of fathers' perspectives and experiences of the development of the father-infant relationship, in fathers who have experienced perinatal mental health difficulties	*N* = 10 fathers of child aged 3–6 years with postnatal mental health difficulties	Anxiety and disordered eating including anorexia (*n* = 2). Postnatal depression and non-birth related trauma (*n* = 1). Depression (*n* = 1) Depression, anxiety and perinatal trauma (*n* = 1) Non-birth related trauma and postnatal depression (*n* = 1) Anxiety (*n* = 2) Birth trauma/PTSD (*n* = 1) Postnatal depression and anxiety (*n* = 1) Self-reported (method of data gathering Not reported)	*Age of participants:* Ranged from 26 to 41 years old *Number of children:* One (*n* = 5) Two (*n* = 4) Three (*n* = 1) *Age of children:* < 1 (*n* = 2) 1 years (*n* = 3) 2 years (*n* = 6) 3 years (*n* = 1) 4 years (*n* = 1) 5 years (*n* = 1) 7 years (*n* = 2) *Marital status:* Married (*n* = 7) Single (*n* = 1) In a relationship (*n* = 2) *Education and/or employment status:* Not reported *Ethnicity/nationality:* White British (*n* = 7) White Irish (*n* = 1) Indian (*n* = 1) White and Asian (*n* = 1)	Social media and snowball sampling	Semi-structured interviews Constructivist grounded theory (CGT; Charmaz, 2014)	Four main themes: 1) Core process: surviving to connecting (two sub themes) 2) View of self as father (three sub-themes) 3) Relationship to self (two sub-themes) 4) Response to distress (two sub-themes)
13.	Hambidge et al. (2021), UK	To explore fathers' perceptions of perinatal support for their mental health problems	*N* = 29 fathers with postnatal mental health difficulties	Postnatal mental health difficulty (*n* = 16) Mental health challenges disclosed: depression, anxiety, stress, borderline personality disorder, bipolar disorder, obsess-compulsive disorder as assessed via study questions regarding symptoms. Medical diagnosis (*n* = 11) Self-reported (*n* = 18)	NB. Characteristics are not listed for whole sample. *Age of participants:* 18–24 (*n* = 4) 25–34 (*n* = 12) 35–39 (*n* = 4) 40–49 (*n* = 4) 50+ (*n* = 2) *Number of children:* Not reported *Age of children:* Not reported *Marital status:* Married (*n* = 18) Co-habiting (*n* = 12) Single (*n* = 4) *Education and/or employment status:* Not reported *Ethnicity/nationality:* White British (*n* = 16) White Irish (*n* = 4) Black British (*n* = 2)	Social media (Twitter, Facebook and LinkedIn); tagged mental health support group for fathers, Make Birth Better and Dad Matters UK.	Qualitative questionnaire (12 questions) created based on guidance from father mental health experts exploring participants' history of mental health, mental health status during pregnancy, birth and postnatal, diagnosis and support received and relationship changes with partner and child as their mental health status changed. Thematic analysis (Braun and Clarke, 2006)	Three main themes: 1) Factors influencing fathers' mental health (four sub-themes) 2) Consequences of poor mental health in fathers (three sub-themes) 3) Solutions to improve fathers' mental health (two sub-themes)

Nine studies focused on fathers with postnatal depression only (Baral and de Guzman, 2021; Dallos and Nokes, 2011; Darwin et al., 2017; Davenport and Swami, 2023; Eddy et al., 2019; Edhborg et al., 2016; Malik, 2025; Pedersen et al., 2021; Schmitz, 2025; see Table 2 for details) and four studies included fathers/non-gestational parents with other mental health difficulties (Atkinson, 2023; Copland and Hunter, 2025; Hambidge et al., 2021; Howat et al., 2023). Diagnoses were verified by a clinician (*k* = 7; Copland and Hunter, 2025; Davenport and Swami, 2023; Hambidge et al., 2021; Howat et al., 2023; Malik, 2025; Pedersen et al., 2021; Schmitz, 2025), self-report (*k* = 6; Atkinson, 2023; Copland and Hunter, 2025; Eddy et al., 2019; Hambidge et al., 2021; Howat et al., 2023; Schmitz, 2025), the Edinburg Postnatal Depression Scale (EPDS; Cox et al., 1987; *k* = 3; Baral and de Guzman, 2021; Dallos and Nokes, 2011; Edhborg et al., 2016), the Gotland Male Depression Scale (GMDS; Rutz et al., 1995; *k* = 2; Baral and de Guzman, 2021; Edhborg et al., 2016), the Patient Health Questionnaire-8 (PHQ-8; Kroenke et al., 2009; *k* = 1; Darwin et al., 2017), the Patient Health Questionnaire-9 (PHQ-9; Spitzer et al., 1999; *k* = 1; Davenport and Swami, 2023), and the Patient Health Questionnaire-15 (PHQ-15; Kroenke et al., 2002; *k* = 1; Darwin et al., 2017).

Most studies reported participant age, number of children, and age of children and marital status, but only four of the 13 (30.8%) studies reported ethnicity (Atkinson, 2023; Darwin et al., 2017; Davenport and Swami, 2023; Howat et al., 2023), and Eddy et al. (2019) had no socio-demographic information due to their data collection method (extraction from blogs). Sample sizes ranged from one to 29. Qualitative data were generated from semi-structured interviews (*k* = 11; Atkinson, 2023; Baral and de Guzman, 2021; Copland and Hunter, 2025; Dallos and Nokes, 2011; Darwin et al., 2017; Davenport and Swami, 2023; Edhborg et al., 2016; Howat et al., 2023; Malik, 2025; Pedersen et al., 2021; Schmitz, 2025), internet forums/blogs (*k* = 1; Eddy et al., 2019) and a qualitative online questionnaire (*k* = 1; Hambidge et al., 2021). Method of analysis varied across the 13 studies. Interpretative Phenomenological Analysis was the most frequently used method of analysis (*k* = 4; Dallos and Nokes, 2011; Davenport and Swami, 2023; Howat et al., 2023; Pedersen et al., 2021), followed by Thematic Analysis (*k* = 3; Copland and Hunter, 2025; Darwin et al., 2017; Hambidge et al., 2021), Content analysis (*k* = 2; Eddy et al., 2019; Edhborg et al., 2016), Colaizzi's seven-step approach (*k* = 1; Malik, 2025), Manen's Hermeneutic Phenomenological Approach (*k* = 1; Schmitz, 2025), Straussian Grounded Theory Design (*k* = 1; Baral and de Guzman, 2021) and Constructivist Grounded Theory (*k* = 1; Atkinson, 2023).

### 3.2 Methodological quality of included studies

Eight studies were assessed, using the CASP, as being of high methodological quality, and five were assessed as moderate (see Table 3), with almost perfect agreement between raters (96.15% agreement, κ = 0.85).

**Table 3:** Methodological quality assessment of all included 13 studies.

Authors, year and location	1. Was there a clear statement of the aims of research?	2. Is a qualitative methodology appropriate?	3. Was the research design appropriate to address the aims of the research?	4. Was the recruitment strategy appropriate to the aims of the research?	5. Was the data collected in a way that addressed the research issue?	6. Has the relationship between research and participants been adequately considered?	7. Have ethical issues beentaken into consideration?	8. Was the data analysis sufficiently rigorous?	9. Is there a clear statement of findings?	10. How valuable is theresearch?	Total
1. Malik (2025)	Yes (1)	Yes (1)	Yes (1)	PA (0.5)	Yes (1)	Yes (1)	Yes (1)	Yes (1)	Yes (1)	Yes (1)	High (9.5)
2. Schmitz (2025)	Yes (1)	Yes (1)	Yes (1)	PA (0.5)	Yes (1)	Yes (1)	PA (0.5)	Yes (1)	Yes (1)	Yes (1)	High (9)
3. Davenport and Swami (2023)	Yes (1)	Yes (1)	Yes (1)	Yes (1)	Yes (1)	Yes (1)	Yes (1)	Yes (1)	Yes (1)	Yes (1)	High (10)
4. Baral and de Guzman (2021)	PA (0.5)	Yes (1)	PA (0.5)	Yes (1)	Yes (1)	No (0)	PA (0.5)	PA (0.5)	Yes (1)	No (0)	Moderate (6)
5. Pedersen et al. (2021)	Yes (1)	Yes (1)	Yes (1)	PA (0.5)	Yes (1)	No (0)	Yes (1)	Yes (1)	Yes (1)	Yes (1)	Moderate (8.5)
6. Eddy et al. (2019)	Yes (1)	Yes (1)	Yes (1)	Yes (1)	Yes (1)	Yes (1)	No (0)	Yes (1)	Yes (1)	Yes (1)	High (9)
7. Darwin et al. (2017)	Yes (1)	Yes (1)	PA (0.5)	Yes (1)	Yes (1)	Yes (1)	Yes (1)	Yes (1)	Yes (1)	Yes (1)	High (9.5)
8. Edhborg et al. (2016)	Yes (1)	Yes (1)	Yes (1)	Yes (1)	Yes (1)	PA (0.5)	Yes (1)	Yes (1)	Yes (1)	Yes (1)	High (9.5)
9. Dallos and Nokes (2011)	Yes (1)	Yes (1)	Yes (1)	No (0)	Yes (1)	Yes (1)	No (0)	Yes (1)	Yes (1)	Yes (1)	Moderate (8)
10. Copland and Hunter (2025)	Yes (1)	Yes (1)	Yes (1)	PA (0.5)	PA (0.5)	No (0)	PA (0.5)	Yes (1)	Yes (1)	Yes (1)	Moderate (7.5)
11. Howat et al. (2023)	Yes (1)	Yes (1)	PA (0.5)	Yes (1)	Yes (1)	Yes (1)	Yes (1)	Yes (1)	Yes (1)	Yes (1)	High (9.5)
12. Atkinson (2023)	Yes (1)	Yes (1)	Yes (1)	Yes (1)	Yes (1)	Yes (1)	Yes (1)	Yes (1)	Yes (1)	Yes (1)	High (10)
13. Hambidge et al. (2021)	Yes (1)	Yes (1)	Yes (1)	PA (0.5)	Yes (1)	No (0)	Yes (1)	Yes (1)	Yes (1)	Yes (1)	Moderate (8.5)
% of included studies rated as “Yes”	92%	100%	69%	54%	92%	54%	54%	92%	100%	92%	

PA, Partially Agree.

High (9–10; green), Moderate (6–8; orange) and Low ( ≤ 5; red) as stipulated by (Butler et al. 2020).

All studies were assessed as having a clear statement of findings and were deemed suitable by the first author for this meta-synthesis because they were exploring the subjective experiences of participants in relation to postnatal depression. Five studies (see Table 3 for details) did not report on discussions about recruitment or explained why the participants selected were the most appropriate as per the “consider” section of the CASP for the question “Was the recruitment strategy appropriate to the aims of the research?”. Dallos and Nokes (2011) did not report how they recruited their sample at all. Two studies did not adequately explore reflexivity or rigor and four did not mention it at all. Five studies did not thoroughly detail how they explained any potential negative effects of the study on the participants as reflected in the scoring to the question “Have ethical issues been taken into consideration?”. Three studies did not justify their research design. Overall, most studies were of high or moderately high methodological quality, with most collecting data in a way that addressed the research issue and analyzed the data in a rigorous and clear manner.

### 3.3 Thematic synthesis

**Figure 2:**
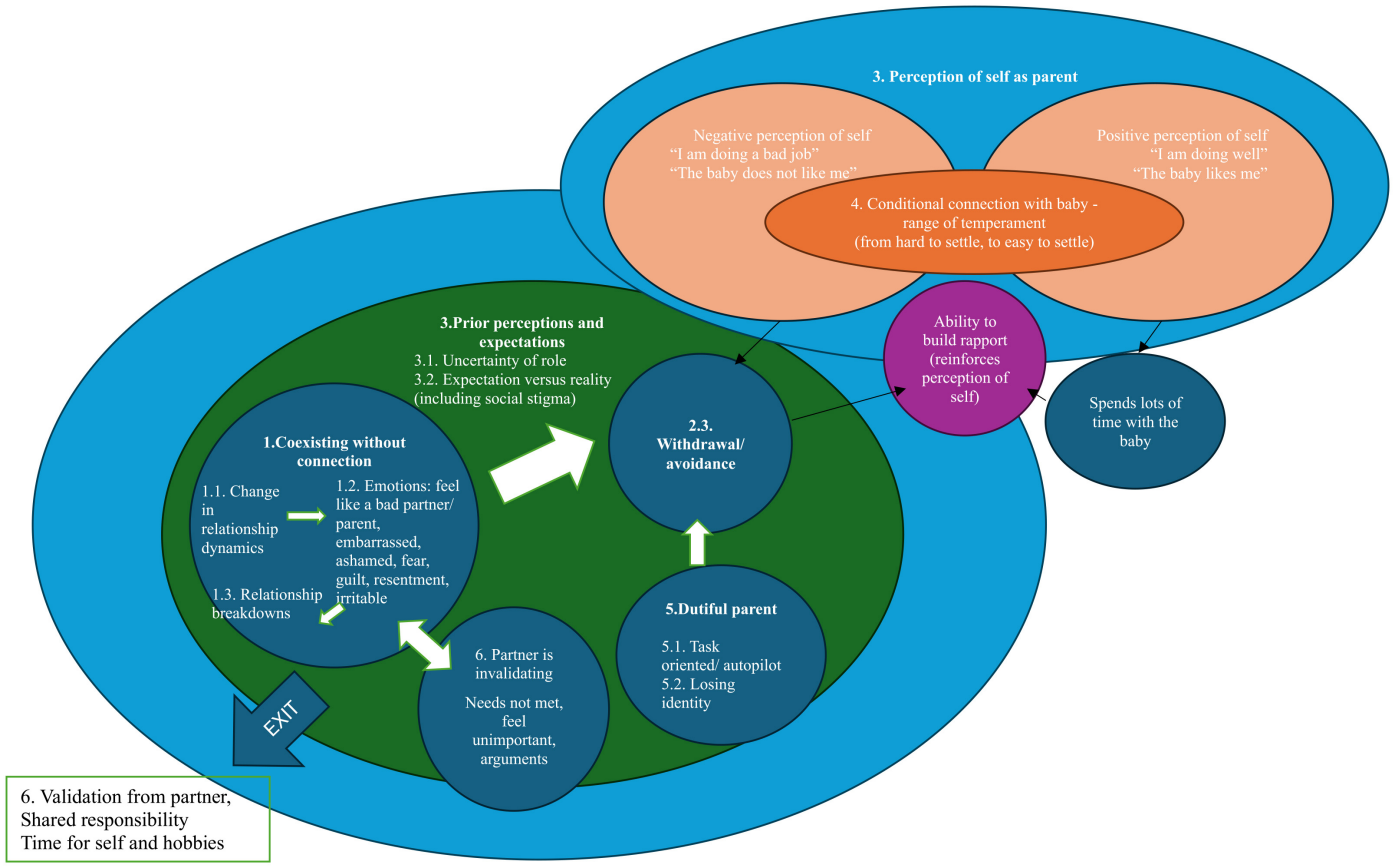
A conceptual model of the themes and subthemes.

Six main themes and nine subthemes were derived from the data: 1) *co-existing without connection* (two sub-themes), 2) *withdrawing from others* (three sub-themes), 3) *prior perceptions and expectations influencing view of self* (two sub-themes), 4) *conditional connection with baby*, 5) *the dutiful parent* (two sub-themes), and 6) *the role of partnership, validation and time for self-care as protective relationship factors*. A conceptual model was developed (Figure 2), which highlights the relationship between the six themes and nine subthemes. When fathers' and non-gestating mothers' prior perceptions and expectations of parenting were different to their reality of parenthood, it appeared to influence their connection to their partner, role as a parent and coping strategy. Fathers' and non-gestating mothers' perceptions of their babies' temperaments also seemed to influence their perceptions of their capability as parents. Spending more time with their baby, rather than using avoidance, allowed fathers to build a stronger rapport and thus increased their perceived self-efficacy as a parent. A matrix of themes highlighted which studies included which themes (see Table 4). When the subtheme related to fathers and non-gestating mothers, this was explicitly stated. Likewise, when a subtheme related only to fathers, only fathers were referred to.

**Table 4:** Matrix of themes from included papers.

Number	Authors, year and location	Theme 1. Co-existing without connection		Theme 2. Withdrawing from others			Theme 3. Prior perceptions and expectations influencing view of self		Theme 4. Conditional connection with baby	Theme 5. The dutiful parent		Theme 6. The role of partnership, validation and time for self-care as protective relationship factors
		Relationship dynamics	Postnatal depression emotions influencing the relationship	Impact of societal norms	Protecting their partner	Avoidance of their family and home as a coping mechanism	Uncertainty about their role as a parent and interactions with their baby	Incongruence between expectations and reality		Task-oriented responsibility	Losing identity	
**1**.	Malik (2025)	✓	✓		✓			✓				
**2**.	Schmitz (2025)	✓	✓	✓	✓	✓	✓	✓	✓	✓	✓	✓
**3**.	Davenport and Swami (2023)	✓	✓	✓	✓	✓	✓	✓	✓	✓	✓	✓
**4**.	Baral and de Guzman (2021)		✓			✓					✓	
**5**.	Pedersen et al. (2021)	✓	✓	✓		✓	✓	✓	✓			✓
**6**.	Eddy et al. (2019)	✓	✓	✓		✓			✓	✓		✓
**7**.	Darwin et al. (2017)	✓	✓	✓	✓	✓		✓	✓		✓	✓
**8**.	Edhborg et al. (2016)	✓	✓		✓		✓	✓	✓	✓	✓	✓
**9**.	Dallos and Nokes (2011)	✓	✓				✓	✓	✓			
**10**.	Copland and Hunter (2025)	✓	✓	✓	✓					✓	✓	✓
**11**.	Howat et al. (2023)	✓	✓	✓	✓		✓	✓	✓	✓		✓
**12**.	Atkinson (2023)		✓	✓				✓	✓	✓		✓
**13**.	Hambidge et al. (2021)	✓	✓	✓	✓		✓	✓			✓	✓

#### 3.3.1 Theme 1: Co-existing without connection

Fathers and non-gestating mothers with postnatal depression described feeling disconnected from their partners following the birth of their baby. Two sub-themes reflected partners living together without connection.

##### 3.3.1.1 Subtheme 1.1: Relationship dynamics

Narratives revealed that relationship dynamics between partners (fathers and biological mothers, or non-gestating mothers and gestating mothers) changed, with fathers and non-gestating mothers with postnatal depression perceiving the biological mother as focusing solely on the baby, leaving them feeling left out. Fathers and non-gestating mothers, therefore, felt unsure of how they fit into the newly formed dyad of mother-baby:

“*Whereas fathers, it's about them, you know, them two over there and me. You feel part of that unit but nonetheless, you're always separated slightly… that's just how it is*” (Darwin et al., 2017, p. 9).

In addition, when the gestational mother breastfed, feelings of jealousy arose:

“*Furthermore, some experienced feelings of rejection, maternal jealousy, and resentment over the bond between their partner and their baby, especially when breastfeeding…* “*I got a bit jealous like I wish I had that kind of bond with him*” (Howat et al., 2023, p. 4).

A change in dynamic was paired with a decline in their relationship with their partner due to poor communication, increased irritability, feelings of invalidation, reduced interaction and physical touch:

“…*we were probably just not really talking or not interacting with each other, we were just kind of existing…*” (Darwin et al., 2017, p. 7).

Fathers who were severely depressed discussed their inability to do anything, for example, not being physically present during interactions with their partners, due to being physically unable to “*get out of bed*” (Davenport and Swami, 2023, pp. 1195–1196), and not being mentally present, thus impacting their ability to maintain a relationship with their partner:

“*In the end I just couldn't function… I wasn't myself. I couldn't even make simple decisions*” (Darwin et al., 2017, p. 6). The relationship also exhibited emotional contagion effects: “*She's been very stressed and when she's stressed, I'm stressed, that's how it goes*” (Darwin at al., 2017, p. 7).

Many participants referenced the long-term impact their depression had on their relationship with their partner, with relationships suffering beyond the postnatal period as a result:

“*My wife seemed to consider me selfish and irresponsible. Even when the bickering ended, the wounds never healed. Our marriage took a fatal hit*” (Eddy et al., 2019, p. 1009).

##### 3.3.1.2 Subtheme 1.2: Postnatal depression emotions influencing the relationship

Having postnatal depression meant some fathers and non-gestating mothers reported feeling invalidated by their partners in relation to their mental health, leading to anger and resentment:

“*I was lonely and alone. I felt that even my wife was ignoring me. She never gave enough importance to my feelings and always suggested that it was all simply low mood*” (Malik, 2025, p. 27).

For some fathers and non-gestating mothers, invalidation, paired with their perception of what society stated their role should be (to support the gestating mother and baby in whatever they needed, offer practical help and be resilient) meant they felt unheard, unseen, and unimportant perceiving that the needs of others had to be met before theirs:

“*mum's had the child let's let make mum better, so even part of my role as a dad felt like, […] you've gotta make sure, you know, mum's happy and mum's getting better and recovering, whatever I'm feeling doesn't matter*” (Atkinson, 2023, p. 28).

Fathers and non-gestating mothers described increased irritability because of depression and invalidation which increased arguments in the couple, or which contributed to fathers becoming “*snappy*” (Darwin et al., 2017, p. 5). When irritable and angry, fathers and non-gestating mothers expressed feeling like a bad partner:

“*I recall getting furious with my partner over trivial things and not knowing why. And I felt wrong about that as well*” (Malik, 2025, p. 29).

#### 3.3.2 Theme 2: Withdrawing from others

This theme, which comprised three sub-themes, highlighted that fathers and non-gestating mothers with postnatal depression withdrew from their families for different reasons including navigating societal norms, protecting their partner and avoidance to cope with their postnatal depression.

##### 3.3.2.1 Subtheme 2.1: Impact of societal norms

Fathers and non-gestating mothers expressed withdrawing from their family through repression of negative emotions and not disclosing how they truly felt due to feelings of shame, embarrassment, fear and guilt:

“*Participants hid their feelings from others to* “*keep up appearances*” *(Sarah), due to not wanting to be a burden, or fearing stigma and dismissal from others*” (Howat et al., 2023, p.5).

Fathers also revealed withdrawing emotionally and physically from their immediate family. Narratives indicated that societal stigma influenced how participants felt: as they were not the parent who gave birth to their baby, participants felt society perceived them as having no reason to be depressed, with the expectation they should not get postnatal depression and that men should remain “*stoic*” (Schmitz, 2025, p. 7):

“*I don't feel I can tell my wife about these feelings. It will make me look weak…*” (Eddy et al., 2019, p. 1007).

##### 3.3.2.2 Subtheme 2.2: Protecting their partner

Participant narratives across the studies indicated fathers and non-gestating mothers suffered in silence and expressed withdrawing to protect their partner's wellbeing. Participants perceived their partner as more fragile than them when they were also struggling in the postpartum period and they did not want to add to their partner's difficulties:

“*She was constantly exhausted and stressed, and I didn't want to make matters worse by bringing up my issues. I felt I needed to appear tough for all of us, but inside, I was crumbling*” (Malik, 2025, p. 27).

In some instances, fathers put on a “façade” and pretending they were not depressed:

“*My behavior still stays the same I'm still very much the same person. I was just unhappy*” (Davenport and Swami, 2023, p. 1195).

Participants compared themselves to their partners who they perceived had a more challenging time than them due to physically recovering from labor, and because the gestating parent tended to be with the baby for a greater amount of time whilst on maternity leave. Thus, participants needed to cope despite struggling:

“*I'm always conscious that [partner]'s got it a lot worse, so I just sort of get on with it*” (Darwin et al., 2017, p. 5).

However, putting their partner's needs before their own added to feelings of invalidation and did not improve their relationship with their partner.

##### 3.3.2.3 Subtheme 2.3: Avoidance of their family and home as a coping mechanism

These feelings coincided with fathers (non-gestating mothers did not report this) using avoidance to cope, spending more time at work or going to the gym to avoid going home and thus spent less time with their families:

“*I try to do longer hours, just get up early and go to bed very late and work as hard as I can all the time*” (Darwin et al., 2017, p. 7).

Fathers avoided going home to cope with their postnatal depression because some found depression impacted them most when they were at home, with their partner and baby. Participants reflected that intentional time apart from their family negatively impacted the family unit:

“*I pulled away from my family and started to spend more time outside of home, socializing and looking for companionship. It nearly destroyed my family*” *(*Eddy et al., 2019, p. 1007).

Other fathers stated that their families had noticed and commented they had withdrawn and spent less time with them, highlighting their avoidance had impacted their family:

“*They said that I have become very serious and that I don't join them anymore in free time*” (Baral and de Guzman, 2021, p. 1086).

#### 3.3.3 Theme 3: Prior perceptions and expectations influencing view of self

This main theme was characterized by fathers and non-gestating mothers who spoke of their and other people's perceptions and expectations of themselves, with uncertainty about how they should interact with their baby and what their “role” should be. Those who strived for perfection seemed to struggle when their reality did not meet their expectation and this discrepancy coincided with feelings of parenting “wrong” which impacted their ability to build a bond with their baby.

##### 3.3.3.1 Subtheme 3.1: Uncertainty about their role as a parent and interactions with their baby

Fathers and non-gestating mothers expressed not knowing what their role as a parent was: ‘

‘*Ah, and I've been struggling in a way to try and find what... what is my role with this child. Um, is it to do as (Esme) does, i.e., feed him, wind him, change his nappies, bath him, clothe him? Do all those things. Everything*” (Dallos and Nokes, 2011, p. 157).

Some participants did not feel like an important member of the family because of this particular uncertainty, suggesting that when the gestational mother takes the role of primary caregiver, it can make the “other” parent feel not needed:

“*I feel totally unimportant [...] what is it, that my role is then? [...] I hoped [...] that we would be equal*” (Pedersen et al., 2021, p. 5).

There was also uncertainty when it came to comforting the baby, with some fathers commenting on not knowing how to do this and feeling guilty as a result. This uncertainty meant that sometimes fathers and non-gestating mothers felt unneeded by their baby and this impacted their bond with their baby:

“*in the middle of the night crying with her [the baby]... apologizing to her... I don't know what to do*” (Schmitz, 2025, p. 5).

##### 3.3.3.2 Subtheme 3.2: Incongruence between expectations and reality

This subtheme encapsulates expectations of the role of being a father. These expectations were taken from society, social media and own beliefs and provided parents with the impression that fathers were meant to be more involved than they were historically. Those who held ideals that they had to be involved described feeling as though they were striving for perfection in a system that was not yet set up for them to take a more active role at the newborn stage (e.g., due to having limited statutory parental leave and having to return to work), particularly when they had postnatal depression:

“*I think a lot of it's to do with social media, like, with other people for—just sharing pictures of like happy families, and everything's great, they're managing fine, and, and then I was like how, what, why is—what am I doing wrong […] it's just, there's expectations of how I should feel vs. how I actually felt*” (Atkinson, 2023, p. 22).

The reality of having postnatal depression meant fathers and non-gestating mothers stated that they could not live up to these expectations, which appeared to result in greater incongruence between expectations and reality and them feeling like a bad parent:

“*Look at my son I'm not even picking up my son I'm not cuddling with him I'm not interacting with him I'm not being the dad I wanted to be I'm failing at that I'm failing as a father…*” (Davenport and Swami, 2023, p. 1196).

#### 3.3.4 Theme 4: Conditional connection with baby

This theme reflected a lack of emotion fathers and non-gestating mothers with postnatal depression had in relation to their babies which sometimes led to strong emotional responses:

“*When I'm personally caring for our son I'm overwhelmed with hate. I hate this baby. I thought my dislike for him would go away and I'd start to bond but it's gotten worse. I hate him*” (Eddy et al., 2019, p. 1008).

Some fathers physically rejected their baby by not being present to care for their baby, and emotionally rejected their baby due to perceptions that the baby was dictating their lives which contrasted with their expectations of their baby fitting into their lives:

“*This child [went] from being something fantastic to be a drag, a major source of irritation in my everyday life*” (Pedersen et al., 2021, p. 5).

Babies who could not be easily consoled were seen by fathers and non-gestational parents with postnatal depression as more challenging to build an emotional connection with:

“*my daughter wouldn't settle and I just started shouting at her… I just can't get her to calm down*” (Davenport and Swami, 2023, p. 1195).

This finding suggested that fathers and non-gestating mothers interpreted crying and fussiness as evidence that their baby did not like them and thus, they perceived themselves as failing when the baby cried. In contrast, bond development was facilitated when fathers and non-gestating mothers perceived the baby as having a positive reaction to them. It is possible that fathers interpreted their baby's positive interactions and responses as indicators that they were doing a good job and were loved by their baby which helped their bond to develop:

“*I just think I'm happy because she's healthy, she's smiling… So I think, well, I must be doing something half right for her to be trotting around as she does, and she's happy with me*” (Darwin et al., 2017, p. 9).

#### 3.3.5 Theme 5: The dutiful parent

This theme, and its two sub-themes, highlights that both fathers and non-gestating mothers were striving to be a “dutiful parent,” by working tirelessly despite their mental health difficulties to complete tasks to fulfill their perceived responsibility as a parent. Participants expressed feelings of losing their pre-child identity and feeling numb. Fathers spoke of no longer having time for themselves and with that, resented their new role as a parent.

##### 3.3.5.1 Subtheme 5.1: Task-oriented responsibility

Participants reported feeling like they acted on “autopilot” and “going through the motions” of changing nappies, feeding and putting their baby to sleep, whilst ignoring their own distress. Fatherhood/motherhood was seen as a task/chore that had to be completed, potentially suggesting that participants perceived caring for their baby as a demand which thus was met with psychological resistance:

“*There is no time for anything else than work and taking care of the infant, I feel like a robot with no choices or happiness in life*” (Edhborg et al., 2016, p. 432).

Participants completed tasks by going through the motions in a robotic way signifying they aimed to do what they perceived was required of them, but without any emotion. Therefore, whilst physically present and fulfilling their role from a physical perspective, this numbness often impacted the emotional element of being a father and non-gestating mother with participants commenting on feeling detached from their baby:

“*Several participants alluded to being physically present but emotionally absent*.” *Jude spoke of times when he was* “*doing whatever [I needed] to do... just the actions*” (Schmitz, 2025, p. 6).

##### 3.3.5.2 Subtheme 5.2: Losing identity

Fathers with postnatal depression perceived that by caring for their child they were losing themselves and having to “*sacrifice all their interests and social life*” (Edhborg et al., 2016, p. 443). The time required to look after a baby was potentially not anticipated with participants reflecting on elements of their lives before the arrival of their baby that they were no longer able to engage with:

“*There's so much that changes. And that you have to give up. And some people say it's not even giving up, but I felt like I was giving up large parts of who I was*” (Copland and Hunter, 2025, p. 6).

Losing oneself and focusing on tasks coincided with some not feeling able to function:

“*In the end I just couldn't function… I wasn't myself. I couldn't even make simple decisions*” (Darwin et al., 2017, p. 6).

Father narratives explored the impact postnatal depression had on their emotions and feeling numb toward everything which ultimately impacted on their ability to form a bond with their baby:

“*Somebody could walk up and punch me in the face, and I would probably just fall over... because I have no... feeling at this point*” (Schmitz, 2025, p. 5).

#### 3.3.6 Theme 6: The role of partnership, validation and time for self-care as protective relationship factors

This theme summarizes factors that helped fathers and non-gestating mothers struggling with postnatal depression repair or maintain positive relationships. Fathers and non-gestating mothers who had a partnership and were able to be “*each other's support system*” (Schmitz, 2025, p. 6) noted improvements in the relationship at a later stage making the couple “*stronger in the long run*” (Howat et al., 2023, p. 4). This finding was symptomatic of a rupture and repair process in which trust and intimacy can be deepened by repairing conflict:

“*There's always been the shared responsibility of if we see the other person getting a bit stressed up by it then the other person takes him away*” (Darwin et al., 2017).

Fathers spoke of their partners recognizing changes in their behavior before they had noticed themselves, suggesting partners could detect symptoms of postnatal depression:

“*[My girlfriend] probably saw the signs before I could [...]*” (Pedersen et al., 2021).

Fathers and non-gestating mothers found it helpful and validating when partners recognized something was wrong and suggested support from services. This finding also suggests partners can be effective at encouraging fathers and non-gestating mothers to seek support:

“*my wife was very good at pointing out in a very gentle way, and supportive way, she was very good at saying look, you, you're struggling with this, and that's okay, it's okay to struggle with it, let's talk about it and let's think about solutions*” (Atkinson, 2023, p. 31).

Self-care and having time to themselves was seen as important by fathers. Fathers perceived that having time for self-care facilitated bonding with their baby. It is possible that having the opportunity to take time for oneself allowed fathers the opportunity to provide all their attention to their baby when they were with them, rather than thinking about other things they could be doing, thus strengthening their attunement with their baby:

“*now I've had time to look after myself, and not have all my attention all the time diverted to something else- cos when I do spend my time with him, I'm enjoying myself and I'm focused on him*” (Atkinson, 2023, p. 28).

## 4 Discussion

This systematic review of 13 studies is the first to synthesize qualitative research exploring fathers' and non-gestating mothers' perceptions of the influence of their postnatal depression on their relationships with their partner and baby. Interestingly, many themes identified in the papers were consistent across fathers and non-gestating women, suggesting both groups of parents experienced similar relational challenges which could be indicative of how they were perceived by others, particularly in maternity services, often feeling invalidated (Greenfield and Darwin, 2024). However, it is important to note that only one study (Howat et al., 2023) included the voices of seven non-gestating women.

The relationship between fathers or non-gestating mothers with postnatal depression and their partner was often described as worsening following the birth of their baby due to changes in the dynamics of their relationship, which were discussed alongside negative emotions. Similar findings have been discussed in couples who did not have postnatal depression (Tufail et al., 2025). However, the current review found not only did the shift in dynamic seem to affect the parental relationships but conflict in relationships was perceived to affect children; according to Zemp et al. (2016), improved parental dyadic coping reduces child behavioral difficulties.

Fathers' and non-gestating mothers' perceptions of their role, and their prior expectations vs. the reality of having a baby, coincided with already heightened emotions in their relationship with their partner. Machin (2015) highlighted the impact the gap between high expectation and reality of fatherhood could have on the father's emotional state in fathers without postnatal depression, with similar findings noted in mothers (Doherty et al., 2025). However, when men aim to be an involved father, they face additional barriers to mothers including societal stigma, economic barriers such as returning to work, and a lack of support from healthcare professionals and government policies (Machin, 2015).

Fathers and non-gestating mothers, who had relational conflict with partners, had disparity between the type of parent they wanted to be when faced with the reality of having postnatal depression. Thus, falling into a role in which they dutifully carried out tasks for their baby and partner, whilst feeling numb to their experience, thus losing their identity and becoming resentful. Smith et al. (2022) explored the experiences of unintended fathers and highlighted similar findings, with men expressing a loss of their identity and negative affect toward their new life. Choi et al. (2005), who explored postnatal depression in women, found similar experiences in relation to the role of being a mother and losing one's own identity. Likewise, Raphael (1975) coined the term *matrescence* to describe a process in which women experience an identity shift, hormonal change and brain structuring during pregnancy and into motherhood. In the current review, carrying out the role of the dutiful parent and changes in the relationship with their partner coincided with withdrawing which maintained the father's position in the family as separate from the mother and the baby, thus increasing jealousy toward the mother-infant dyad. A qualitative study exploring the emotions of men without postnatal depression during their transition to fatherhood reported a similar finding, with men expressing feelings of exclusion due to the bond between mother and baby (Solberg et al., 2023). When considering the theme pertaining to expectation vs. reality of fatherhood, it is important to consider the intended role of fathers prior to fatherhood and mothers prior to motherhood. Raphael-Leff (2010) explored parental orientations in the context of parenting and bonding and concluded that different maternal and paternal orientations when paired can mitigate postnatal depression in mothers. According to (Raphael-Leff 2010), men who desire to have sole care of the baby can moderate postnatal depression in mothers who view motherhood as a learned process; however, any other paired mother-father orientation was believed to result in maternal postnatal depression. It would be interesting to explore orientation in the context of same-sex couples, and/or paternal postnatal depression.

Fathers and non-gestating mothers with postnatal depression perceived their ability to form a bond with their baby as more challenging if they perceived their baby as having a challenging temperament (crying frequently and not easily settled). Goldberg et al. (2002) explored fathers without postnatal depression and their level of engagement with their infant and found similar results with fathers of children perceived as having difficult temperaments, having reduced responsiveness, involvement, and affection toward their children. Moreover, Freed and Tompson (2011) suggested that parents with high parental locus of control believed their parenting behaviors could impact their child's development. The current review suggested that the participants had a high internal locus of control which led to internalizing blame for their baby's reactions, and perceiving the baby to not like them, leading them to the conclusion that they were a bad father or mother. Likewise, when fathers perceived positive interactions with their babies, fathers interpreted this as them doing a good job, that their baby did like them, thereby increasing the bond. Saharoy et al. (2023) cite similar findings with mothers with postnatal depression whereby the characteristics and behaviors of the baby could aggravate or alleviate the effects of postnatal depression on maternal care.

Of note, paternal postnatal depression symptoms are different to women's symptoms, often including restlessness, appetite changes, irritability, impaired concentration and work performance, and social isolation (Berg and Ahmed, 2016), in addition to anger and substance use (Epifanio et al., 2015; Mangialavori et al., 2021). In the current review, fathers expressed using avoidance as a coping strategy, possibly related to the different symptoms they present with. However, when fathers used avoidance as a coping strategy and thus spent less time with their baby, there were fewer opportunities to have positive interactions with their baby. As a result, a negative feedback loop might have reinforced the perception that their baby did not like them, and reduced their self-efficacy so fathers withdrew further. This finding highlights the importance of increasing fathers' self-efficacy, which Carbone et al. (2025) has suggested is beneficial to improve child mental health. Similarly, fathers with increased caregiving and greater time spent with their baby have increased oxytocin and greater amygdala activations, specifically in the superior temporal sulcus, suggesting that greater exposure with their baby facilitates biological and neurological bonding such as an increased ability to interpret their baby's social cues and signals (Abraham et al., 2014). Therefore, it is possible that who spend more time with their babies have a greater bond due to physiological changes, thus withdrawing from babies reduces opportunities to develop a bond. It is important to consider that avoidance of the family home is not as feasible for gestational women with postnatal depression due to physical recovery from birth and often being the primary caregiver on maternity leave and feeling confined to their homes (Taylor et al., 2021). However, it is common for gestational women with postnatal depression to want to isolate themselves, by remaining in the house without their baby and others (Highet et al., 2014). It is interesting that this finding was not noted in non-gestational women, which potentially highlights differences in the way men and women cope with difficulties in the family home, with men more likely to utilize avoidance as a coping strategy (Berke et al., 2018).

### 4.1 Clinical implications

As the current review highlights the need to support fathers and non-gestating mothers with postnatal depression to minimize the perceived influence on their relationships with their partner and baby, several recommendations are made (see Table 5). Interestingly, some fathers and non-gestating mothers described their partners noticing a change in mood, validating their experiences and encouraging them to seek support. However, there are known barriers to men seeking psychological support, such as emotional avoidance, poor knowledge of available services, cultural norms about fatherhood and masculinity, and stigma in relation to mental health (Mancinelli and Filippi, 2025). Thus, when partners did not validate or notice changes in their partners, fathers were less likely to seek support. Therefore, fathers and non-gestating parents should aim to be aware of their own barriers to seeking support for postnatal depression. Postnatal depression in fathers looks different to postnatal depression in biological mothers, with fathers becoming angry; whilst continuing to carry out their responsibilities, they also seek solace in solitude and withdraw from those around them (Chen et al., 2023). Fathers and non-gestating parents should, therefore, be aware of the potential influence of postnatal depression on their relationship with their partner and baby.

**Table 5:** Recommendations for services.

Area of system	Recommendation
Fathers and non-gestating parents	• To be aware of the potential influence of postnatal depression on their relationship with their partner and baby. • To be aware of their own individual barriers to seeking support for their postnatal depression.
Health care professionals (e.g., GP's, midwives)	• To routinely screen fathers and non-gestating mothers for postnatal depression. • To signpost fathers and non-gestating mothers to services to help them with their postnatal depression. • To implement screening in a way that overcomes known barriers.
Mental health services and commissioners	• To ‘think family' and include fathers/non-gestating parents in therapy with gestational mothers where consent is provided, and there is a clear rationale. • To fund therapeutic services for fathers and non-gestating parents who have postnatal depression.

Services need to explore ways to overcome screening barriers related to lack of a standardized measure and men's reluctance to seek support for their mental health (Reay et al., 2023): for example, utilizing screening apps which are readily accessible (Eisner et al., 2025). Screening measures commonly used by healthcare professionals with biological mothers (Cox et al., 1987) do not currently capture the male presentation of postnatal depression. Relatedly, a modified Delphi approach with 14 international expert panelists was unable to reach a consensus on the best screening tool for postnatal depression in men; however, it was noted that the EPDS was the most frequently utilized tool (Freitas et al., 2016). Therefore, it is recommended that the EPDS could be utilized whilst a specific screening tool for postnatal depression in men is being developed.

In the UK, clinical services are encouraged to think holistically about the family; for example, in their guidance, Darwin et al. (2021) stressed the importance of considering the whole family in perinatal services. Likewise, Canada (Reproductive Mental Health Program and Perinatal Services BC, 2024), Scotland [Scottish Intercollegiate Guidelines Network (SIGN), 2012] and Australia (Highet and the Expert Working Group and Expert Subcommittees, 2023) recommend that perinatal mental health services consider assessing and addressing the needs of partners. Additionally, in the UK the British Psychology Society issued guidance which recommends including partners in therapy if the mother consents (Peterkin et al., 2024). A recent example of this in practice was implemented in a specialist perinatal mental health service in the UK which developed and found promising results using attachment narrative therapy with families, based on the acknowledgment that babies are raised in families and systems, not just mother-baby dyads (Farrer et al., 2025). However, family work in specialist perinatal mental health services in the UK is not yet fully implemented, potentially due to the restrictions placed on these services due to lack of commissioning for supporting fathers. Moreover, fathers have expressed that General Practitioners (GP) (Baldwin et al., 2019) and primary care are not adequately provisioned or trained to support men in the perinatal period (Rowe et al., 2013). As such, fathers and non-gestating mothers first may need better and more consistent screening for postnatal depression to identify those in need of support (Mancini et al., 2025). Therefore, it is imperative for commissioners to fund provision and resources to support the mental health of fathers and non-gestational parents in the postnatal period, in either primary or secondary services, and in specialist perinatal mental health services to support men via signposting to relevant mental health services, charities and resources (Darwin et al., 2021).

### 4.2 Strengths, limitations, and future research

The search was systematic and comprehensive across nine databases. Double screening was conducted, with 25% of papers at the abstract and title screening stage, and 30% at the full text screening stage. The review search generated non-English language papers, although none met the eligibility criteria at full text screening. This approach minimized publication and geographical biases, ensuring higher methodological rigor in the search and review process, and minimizing the omission of crucial data (Walpole, 2019). Although the first author, who was the main screener, was monolingual in English, EndNote provided an English version of the abstract and title, irrespective of the language in which the full text was written. At full text screening, the author used Google Scholar to translate from Spanish and French to English, as necessary. Google Scholar has been found to be 96% accurate when translating English to Spanish for patient-specific discharge instructions (Kong et al., 2026). Additionally, Balk et al. (2013) assessed the accuracy of Google Translate when used for data extraction from other languages into English and found that articles in Spanish were translated with high accuracy, as were French ones. The review also utilized a recognized quality measure, the CASP, which has been found to be a good measure for transparency of research practice and reporting standards (Long et al., 2020). Included studies were from six countries which is a strength of the review because different cultural perspectives will have been considered, although it would be beneficial to have research from additional non-western countries included.

A limitation is that only the first author extracted data and, therefore, the accuracy and consistency of data extracted cannot be commented on (see Calderon-Martinez et al., 2025). However, the inclusion of papers, the data and results were discussed during meetings with the review team who had different backgrounds, expertise and experiences. This triangulation of analyses through discussion was enhanced and facilitated assessment of rigor (Lee et al., 2014). Additionally, to support low-cost translations the first author utilized Google Translate and thus there is a possibility that some translations might not have been completely accurate (Rockliffe, 2022).

Furthermore, the review included four papers of parents with diagnoses that were not solely postnatal depression. A known challenge in qualitative research is the conflict between anonymizing data to protect participants and retaining enough contextual information to accurately interpret the data (Thomson et al., 2005). Due to the paucity of literature, it was decided to include papers which included participants with postnatal depression and other mental health diagnoses. However, in retrospect this was a significant limitation, because it proved to be impossible in some cases to determine who the quotes belonged to due to anonymization, and, therefore, it is possible that some findings were taken from participants with a different mental health difficulty. Although the 13 papers utilized seven different methods of analysis, which could lower confidence in the review findings (Munthe-Kaas et al., 2018), most of the included papers generated themes through inductive coding which enabled the author to reduce varied text into summaries, ascertain links between objectives and findings from the data, and develop models/theories (Thomas, 2006).

Of the included papers, only 30.8% assessed/reported the ethnicity of the participant. Future research should aim to include this information to support interpretation across different ethnic groups and to better inform clinical implications for different ethnic populations given the different experiences faced by different groups, particularly in perinatal services (Vo et al., 2024). Additionally, researchers could explore methods of screening fathers and non-gestating mothers, taking into consideration known barriers such as reduced contact with perinatal staff such as midwives and health visitors (Mancinelli and Filippi, 2025).

Finally, given the qualitative nature of the review, the review did not consider wider context in relation to the health of the baby, such as life-limiting conditions diagnosed before or after birth (e.g., trisomy 18), which may represent important risk factors for postnatal depression and relational dynamics (Dahò, 2020). Future studies may also wish to consider stillbirth and neonatal deaths (e.g., SIDs) when exploring the relationships solely between fathers/non-gestational parents and their partners.

## 5 Conclusion

This current systematic review found that fathers' and non-gestating mothers' postnatal depression appeared to influence their relationships with their partner and baby, with tensions present in both relationships. Whilst some of the findings underscored the similarities between the experiences of fathers, non-gestational parents and mothers, this review also revealed that only fathers utilized avoidance as a coping strategy, which could be related to the different symptoms they experienced in postnatal depression and given the societal stigma they faced in relation to not being expected to have postnatal depression. The review also found a large crossover between fathers' and non-gestational mothers' experiences relating to the influence postnatal depression appeared to have on their relationships with their partner and baby, which could be indicative of the experiences they share in maternity services and feeling outcast. These novel findings point toward the need for maternity, GP and perinatal mental health services to consider the health and wellbeing of both parents during the perinatal period.

## Data Availability

The original contributions presented in the study are included in the article/supplementary material, further inquiries can be directed to the corresponding author.
